# The Biophysical Characterisation and SAXS Analysis of Human NLRP1 Uncover a New Level of Complexity of NLR Proteins

**DOI:** 10.1371/journal.pone.0164662

**Published:** 2016-10-11

**Authors:** Luigi Martino, Louise Holland, Evangelos Christodoulou, Simone Kunzelmann, Diego Esposito, Katrin Rittinger

**Affiliations:** 1 Molecular Structure of Cell Signalling Laboratory, The Francis Crick Institute, 1 Midland Road, London, NW1 1AT, United Kingdom; 2 Structural Biology Science Technology Platform, The Francis Crick Institute, 1 Midland Road, London, NW1 1AT, United Kingdom; Virginia Polytechnic Institute and State University, UNITED STATES

## Abstract

NOD-like receptors represent an important class of germline-encoded pattern recognition receptors that play key roles in the regulation of inflammatory signalling pathways. They function as danger sensors and initiate inflammatory responses and the production of cytokines. Since NLR malfunction results in chronic inflammation and auto-immune diseases, there is a great interest in understanding how they work on a molecular level. To date, a lot of insight into the biological functions of NLRs is available but biophysical and structural studies have been hampered by the difficulty to produce soluble and stable recombinant NLR proteins. NLRP1 is an inflammasome forming NLR that is believed to be activated by binding to MDP and induces activation of caspase 1. Here, we report the identification of a soluble fragment of NLRP1 that contains the NACHT oligomerization domain and the putative MDP-sensing LRR domain. We describe the biophysical and biochemical characterization of this construct and a SEC-SAXS analysis that allowed the calculation of a low resolution molecular envelope. Our data indicate that the protein is constitutively bound to ATP with a negligible ability to hydrolyse the triphosphate nucleotide and that it adopts a monomeric extended conformation that is reminiscent of the structure adopted by NLRC4 in the inflammasome complex. Furthermore, we show that the presence of MDP is not sufficient to promote self-oligomerization of the NACHT-LRR fragment suggesting that MDP may either bind to regions outside the NACHT-LRR module or that it may not be the natural ligand of NLRP1. Taken together, our data suggest that the NLRP1 mechanism of action differs from that recently reported for other NLRs.

## Introduction

Innate immunity is an ancient mechanism of defence common to both animals and plants, and comprises a large number of pattern recognition receptors (PRRs) [1] that sense the presence of microorganisms by recognising pathogen-associated molecular patterns (PAMPs) [2,3]. PRRs represent the first line of defence and are found expressed on the surface or in the cytoplasm of immune cells, or secreted in tissue fluids [4]. To date, a number of families of proteins with characteristics of PRRs have been identified and include Toll-like receptors (TLRs) and the C-type lectin receptors (CLRs) that are membrane bound, and the NOD-like receptors (NLRs), RIG-I like receptors (RLRs) and the AIM2-like receptors (ALRs) that are expressed as soluble receptors in the cytoplasm [3–5].

All NLRs have a conserved tripartite domain architecture that consists of an N-terminally located effector domain, followed by a central NACHT domain and a C-terminally located ligand sensing domain [6]. The effector domain is usually a member of the death domain super-family of protein-protein interaction domains, specifically CARD or pyrin domains and has the ability to interact with downstream effectors to activate specific signalling pathways [7,8]. The NACHT domain is an ATP-binding domain that belongs to the super-family of the AAA+ ATPases and mediates NLR oligomerization. Structurally it consists of three distinct regions: a nucleotide binding domain (NBD), a winged helix domain (WH) and a super-helical domain (SH) [6,9]. The sensing domain is usually made of a number of leucine rich repeats (LRR) which are believed to sense specific PAMPs [10,11]. The current model for the mechanism of action of NLRs assumes that they are present in the cytosol in an inactive monomeric state. Upon detection of a given PAMP by the LRR, an ATP-dependent conformational change of the NACHT domain promotes the oligomerization of the receptor, resulting in activation of different signalling pathways [9]. Inflammasome-forming NLRs, like NLRP1 and NLRP3, bind to adaptor protein ASC which in turn interacts with pro-caspase 1 forming the inflammasome complex [12]. This molecular structure promotes the maturation of caspase 1, which is required for processing of pro-inflammatory cytokines like interleukin-1β (IL-1β) and interleukin-18 (IL-18) [13–15]. On the other hand, non-inflammasome-forming NLRs can activate a number of different signalling pathways including those mediating activation of nuclear factor κB (NF-κB), mitogen-activated protein kinase (MAPK) and the type I interferon (IFN) response [16]. Two of the best characterised non-inflammasome-forming NLRs are NOD1 and NOD2, which undergo muramyl dipeptide (MDP)-induced oligomerization, allowing interaction with the CARD-containing kinase RIP2 (RIPK2) to promote activation of NF-kB and transcription of pro-inflammatory genes [17,18]. Given their central role in innate immunity, it is not surprising that malfunction of NLRs is linked to a number of autoimmune disorders. For example, defects in NLRP1 are linked to vitiligo and Addison’s disease while defects in NLRP3 can lead to type 2 diabetes, systemic lupus erythematosus, rheumatoid arthritis and Alzheimer’s disease [19]. On the other hand, NOD1 and NOD2 malfunction is linked to celiac disease, Crohn’s disease and Blau syndrome [20]. Moreover, numerous reports have suggested an association between NLR malfunctioning and cancer and cardiac diseases [21–24].

Until recently, the mechanism underlying oligomerization of NLRs was thought to resemble that of the apoptosome, where interaction with cytochrome C induces oligomerization of Apaf1 molecules into a ring-shaped complex [25,26]. However, recent structural work from three different groups on the NAIP2-NLRC4 and NAIP5-NLRC4 inflammasome oligomerization now suggest a different mechanism. These studies have unveiled a complex prion-like oligomerization mechanism for NLRC4, where one molecule of NAIP2 or NAIP5 is able to catalyse the oligomerization of NLRC4 into either a wheel-like or into a fibre-like structure [27–29].

NLRP1 possesses a distinctive domain organisation with two extra domains at the C-terminus, a FIIND and a CARD domain that are not conserved across the NLR-family [30]. Interestingly, the FIIND domain was reported to be an important regulatory motif that performs auto-proteolysis to activate caspase 1 through an alternative mechanism involving ASC and the C-terminal CARD domain of NLRP1 [31,32]. Human NLRP1 has been proposed to recognise MDP and a recent report has suggested that, *in vitro*, MDP and ATP are sufficient to promote NLRP1 oligomerization [33]. To date, only the structures of isolated CARD, pyrin and LRR and domains of NLRP1 have been solved [34–36]; therefore one of our main goals was to gain structural information on the entire protein. However, in our hands, the full length protein was not sufficiently stable and all attempts to obtain pure and homogeneous recombinant samples were hampered by proteolytic degradation of the N- and the C-termini of the protein. In contrast, a construct containing the NACHT and LRR domains was stable and could be purified to homogeneity. We characterised the ability of this construct to bind to the activator molecule MDP and hydrolyse nucleotides and we investigated its oligomeric state and overall shape by Small Angle X-ray Scattering (SAXS). Our data show that the protein fragment containing the NACHT and LRR domains is monomeric in solution, ATP-bound and does not show any measurable nucleotide hydrolysis activity. Furthermore, we found no evidence of increased hydrolysis rate and/or propensity to form high molecular species in the presence of MDP strengthening the suggestion, already made by others [30,34], that MDP may not bind to the NACHT-LRR module and may not be the natural activating molecule for NLRP1. Our data suggest that the mechanism of activation of NLRP1 differs from that proposed in the past and from the recently characterised mechanism of action of NLRC4.

## Material and Methods

### Protein expression in *E*.*coli* and in insect cells

All human NLRP1 constructs were amplified by PCR using a full length human NLRP1 cDNA as template (Imagenes). The protein constructs to be expressed in *E*. *coli* were cloned into pET47b and/or pET49b (Merck Millipore) plasmids to generate N-terminal His_6_- and/or GST-fused constructs. The protein constructs to be produced in insect cells were cloned into pIEX-Bac3 plasmid to generate N-terminal His-fused proteins. All the cloning was performed using the Ligation Independent Cloning (LIC). Plasmids were sequence-verified by DNA sequencing (http://www.sourcebioscience.com/). To test expression and solubility of the human NLRP1 constructs in *E*. *coli*, transformed BL21-Gold(DE3) were induced at 18°C by 0.5 mM IPTG for about 16 hours. Cells were lysed by sonication in 50 mM HEPES pH 7.5, 300 mM NaCl, 1 mM MgCl_2_, 5% glycerol, 1 mM PMSF. The supernatants were loaded onto pre-equilibrated columns containing Ni-NTA-beads or Glutathione Sepharose and purification was carried out following the manufacturer’s instructions. To test expression and solubility of human NLRP1 constructs in insect cells, baculoviruses were prepared by co-transfecting *Sf9* cells with pIEX-Bac3-NLRP1 plasmids and linearised BAC10:KO_1629_ [37], *Sf9* cells were cultured at 28°C in SF900 II serum-free medium (Invitrogen). Expression of the NLRP1 protein constructs was tested by infecting 25 ml insect cells (~2 x 10^6^ cells/ml) with 50 μl of the high titre virus stock for 48 hours. The cells were pelleted and were re-suspended in 50 mM HEPES pH 7.5, 300 mM NaCl, 5 mM MgCl_2_, 10% glycerol, 10 mM imidazole and protease inhibitors (Roche cOmplete EDTA Free), followed by addition of 0.1% Triton X-100 and 2 μg/μl DNase I. Lysed cells were clarified at 5000 rpm, the supernatant was loaded onto columns pre-equilibrated with 150 μl of 50% Co-Talon resin (Clontech-Laboratories), and purification was carried out following the manufacturer’s instructions.

### Large scale protein purification of human NLRP1 constructs from insect cells

Soluble NLRP1 constructs were scaled up by infecting about 2.3 L of Sf9 cells at 2.0 x 10^6^ cells/mL density with about 4–7 mL of high-titre NLRP1-baculovirus for 48 hours. All soluble constructs were purified using a three-step protocol including a metal affinity capture (Co-Talon resin—Clontech-Laboratories), followed by an ion-exchange (Resource S, GE-Healthcare Life Sciences) and a gel filtration step (Superdex S200 XK16/60). The final sample in 25 mM MES pH 6.0, 200 mM NaCl, 5 mM MgCl_2_, 2 mM DTT, was concentrated to 5–8 mg/mL.

### Circular Dichroism spectroscopy

Far-UV CD spectra were recorded between 260 and 200 nm on a JASCO spectrophotometer. The protein solutions were prepared in 25 mM MES pH 6.0, 100 mM NaCl, 0.5 mM TCEP with a final concentration of 0.15 mg/mL. The spectra were acquired at 20°C, with a scan speed of 20 nm/min, 25 accumulations and a path length of 0.1 cm. A spectrum for the buffer was recorded and subtracted from the protein spectra. Thermal unfolding experiments were recorded by following the change in CD signal at 222 nm as function of the temperature. The temperature range investigated was between 2 and 90°C and the temperature was increased with a speed of 2°C/min.

### Determination of the nucleotide bound to NLRP1 by HPLC

To determine the nature of the nucleotide bound to NLRP1 an HPLC analysis was carried out. To 100 μl of recombinant purified NLRP1 at a concentration of about 30–50 μM, 10% perchloric acid (HClO_4_) was added, followed by addition of 15 μl of 4 M sodium acetate. The samples were clarified by centrifugation at 13.000 rpm for 5 min to remove the precipitated protein and 100 μl of the supernatant was injected on to the HPLC. Samples were run on a Partisphere-SAX column (125/4.5 mm, Whatman), with 0.45 M (NH_4_)_2_HPO_4_, adjusted to pH 4.0 with HCl, as mobile phase. The nucleotide content was calculated from the peak areas by comparing to nucleotide standards containing a mixture of 50 μM ADP and 50 μM ATP, prepared in the same buffer as the NLRP1 solutions.

### ATPase activity assay

The malachite green assay (ATPase assay kit, Innova Biosciences) was used to evaluate the ATPase activity of NLRP1. The assay was performed by incubating purified recombinant NLRP1 constructs with ATP and measuring the concentration of free phosphate released in solution upon ATP hydrolysis. The protein concentration used in the assay was 0.1 mg/mL in 25 mM Hepes pH 7.0, 150 mM NaCl, 1mM MgCl_2_ and 1 mM DTT while the initial ATP concentration was 1 mM. The assay was performed at room temperature for 1 hour and the concentration of free phosphate in solution was monitored before adding ATP and after 5, 20, 40 and 60 minutes of incubation. Free phosphate in solution was detected by adding green malachite reagent and by measuring the UV absorption at 620 nm. The assay was also repeated in presence of 0.1 mg/mL MDP. A positive control experiment was also performed using a solution of DnaK at 0.1 mg/mL in presence of 1 mM ATP.

### Small-angle X-ray scattering

SEC-SAXS data for the NLRP1 protein fragment spanning residues 227 to 990 were collected at the Diamond Light Source on beamline B21. In line SEC-SAXS was performed using an Agilent 1200 HPLC system equipped with a 2.4 mL Superdex S200 (GE Healthcare) column. Data were recorded on a Pilatus 2M detector with a fixed camera length of 4.014 m and 12.4 keV energy allowing the collection of the angular range q between 0.0038–0.42 Å^-1^. Samples at two different concentrations (2 mg/mL and 6 mg/mL) were loaded onto the size exclusion column previously equilibrated in 25 mM MES pH 6.0, 500 mM NaCl, 5 mM MgSO4, 1 mM TCEP. The primary reduction of the SAXS data was performed using ScÅtter (http://www.bioisis.net/), the data processing was carried out with ATSAS (http://www.embl-hamburg.de/biosaxs/software.html) to obtain the radius of gyration (Rg), the maximum particle dimension (Dmax), the excluded particle volume (V_p_) and the pair distribution function (P(r)) [38]. A low resolution three-dimensional *ab initio* model for NLRP1(227–990) was generated by the program DAMMIF [39], averaging the results of 20 independent runs using the program DAMAVER [40]. CRYSOL [41] was used to compare crystal structures of NLRC4 in two conformations (PDB 3jbl and PDB 4kxf) with experimental scattering profiles. The *ab initio* model was overlapped to the two crystal structures of NLRC4 using SUPCOMB and the SAXS-derived envelope was rendered with PyMOL.

## Results

### Primary sequence analysis of NLRP1

**Fig 1:**
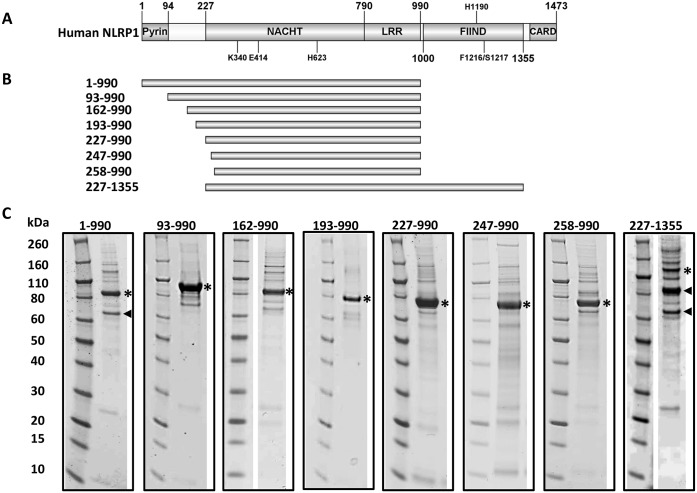
Domain organisation and expression screen of human NLRP1. (A) Human NLRP1 domain organisation; black lines indicate some of the key residues reported to be important for protein function. K340 and E414 belong to the Walker A and Walker B motifs, respectively and are important for ATP processing. H623 is a conserved residue across all the NLRs, the correspondent residues in NLRC4 and in Apaf1 are involved in stabilising the ADP-bound conformation. H1190, F1216 and S1217 are reported to be important for the auto-proteolysis of the FIIND domain. (B) Schematic representation of the soluble constructs produced in insect cells. The boundaries of each construct are indicated on the left. (C) SDS-gels of the recombinant proteins from insect cells after the first metal affinity purification step. A black star indicates the protein of interest and a black arrow heads indicates proteolytic degradation products.

To identify regions of primary sequence conservation between NLRP1 and other NLR proteins, a multiple sequence alignment was performed. The sequences of the 22 NLRs from *Homo sapiens*, two NLRP1 sequences from *Mus muculus* and the NLRP1 sequence from *Rattus norvegicus* were aligned using the Clustal Omega server. As expected, the primary sequences were found to be divergent at the N- and C- termini but two main regions of conservation, common to all sequences, corresponding to the NACHT and the LRR domains, were identified (S1 Fig, red box). A second region of conservation, corresponding to the FIIND domain and the C-terminal CARD, was also clearly identifiable in the NLRP1 sequences (S1 Fig, green box). The regions of conservation identified in this alignment combined with information available in literature were used to define the domains boundaries of human NLRP1. On the basis of this analysis the domain organisation of human NLRP1 is as follows: the pyrin domain spans residues 1–94, the NACHT domain residues 227–790, the LRR domain residues 790–990, the FIIND domain residues 1000 up to 1355 and the C-terminal CARD spans residues 1379 until 1473 (Fig 1A). Notably, the long linker between the pyrin and the NACHT domain is not conserved across NLRs and a secondary structure prediction suggests that this region is likely to be flexible.

### Expression screen to identify soluble fragments of human NLRP1

**Fig 2:**
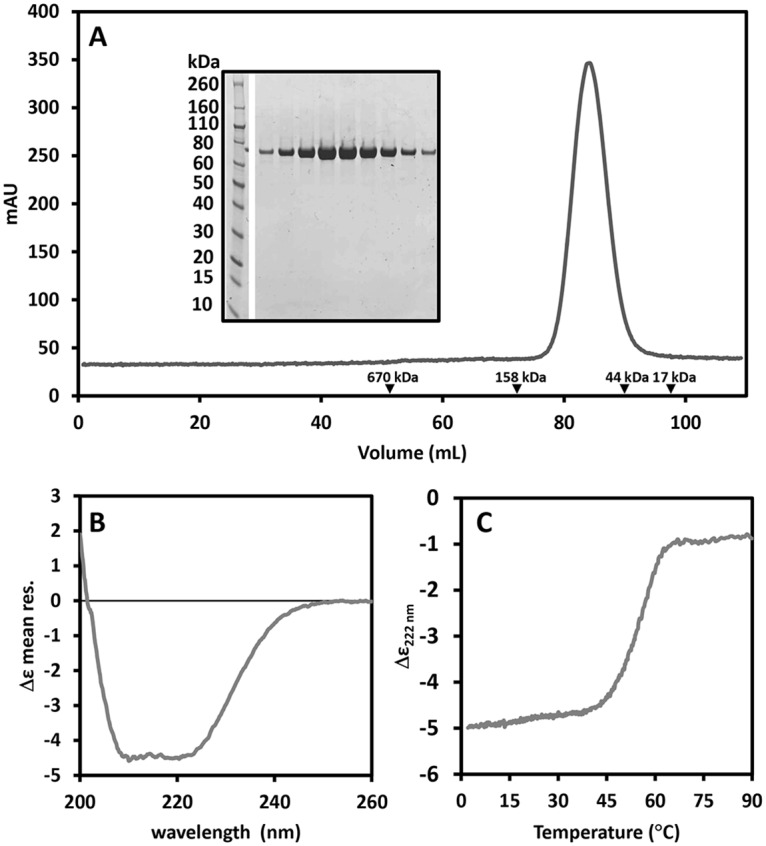
Preliminary biophysical characterisation of recombinant NLRP1(227–990). (A) Size exclusion chromatography profile of the construct NLRP1(227–990) on a S200 XK16/60 column. Black arrow heads indicate the retention volumes of molecular weight standards. The sample migrates as a single species with an apparent molecular weight between 44–158 kDa. (B) Far-UV circular dichroism spectrum of NLRP1(227–990). (C) Thermal unfolding of NLRP1(227–990) obtained by following the CD signal at 222 nm as a function of the temperature increased at 2°C/min, the value of the mid-point transition is 54.3±0.5°C.

To identify soluble and stable constructs of human NLRP1, a number of deletion mutants were designed and tested for expression, both in *E*.*coli* and insect cells (S1 Table). No constructs spanning the pyrin or the CARD domains in isolation were tested because their structures are known. Although a number of constructs for isolated NACHT, LRR and FIIND domains were tested for expression in *E*.*coli*, only the LRR domain, the structure of which was recently reported [34], was found to be soluble. However, a number of longer constructs containing multiple domains were found to be soluble upon expression in insect cells (S1 Table and Fig 1B and 1C). All soluble constructs were produced and purified on a large scale. In our hands, the full length protein and construct NLRP1(227–1355) were very unstable and prone to proteolytic degradation. This was not unexpected as it has been shown that the FIIND domain is capable of auto-proteolysis at residues 1216/1217 [31]. Similarly, construct NLRP1(1–990) containing the pyrin, NACHT and LRR domains also showed severe proteolytic degradation and a tendency to aggregate. On the other hand, a remarkable improvement was observed upon deletion of the N-terminal pyrin domain and the C-terminal FIIND and CARD domains yielding good quantities of homogenous samples. The construct producing the highest yield of stable protein spans residues 227 up to 990 and contains the NACHT-LRR domain, indicated here as NLRP1(227–990) (Mw 89.5 kDa), which runs on a size exclusion S200 XK16/60 column as a single peak with an apparent molecular weight between 44 and 158 kDa. This indicates the presence of a single monomeric species in solution (Fig 2A), without degradation or post-translational modifications as confirmed by mass spectrometry (data not shown). Furthermore, the far-UV CD spectrum recorded at 20°C, shows that the protein is well folded with an overall content of α-helix and β-sheet predicted to be around 47% and 10%, respectively (Fig 2B) and melting temperature of approximately 54°C (Fig 2C). Taken together, those data suggests that the construct spanning residues 227–990 is a well folded protein that is amenable to biophysical studies.

### Identification of nucleotide bound to human NLRP1 and characterisation of its ATP-hydrolysis activity

**Fig 3:**
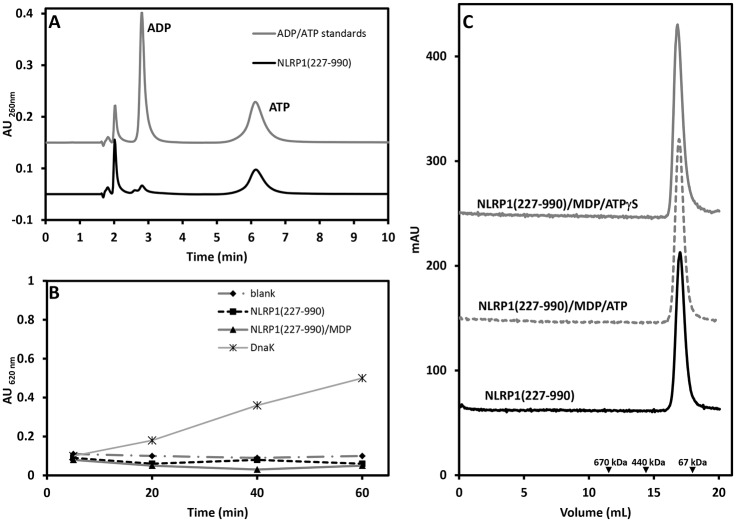
ATP hydrolysis and oligomerization assay of NLRP1(227–990). (A) HPLC chromatogram showing the profile of a solution of ADP and ATP standards run on an ion exchange Partisil SAX column (grey solid line), retention times for ADP 2.81 minutes and ATP 6.12 minutes. The sample prepared by unfolding of NLRP1(227–990) contained mainly ATP (>85%) (black solid line). The peak with a retention time of about 2 minutes is background from the buffer. (B) Malachite green assay performed on a solution of NLRP1(227–990) in the presence of ATP 1mM (black squares) and in the presence of ATP 1mM and MDP 0.1mg/mL (black triangles). In both cases the concentration of protein was 0.1 mg/mL and the experiment was performed at room temperature for 1 hour. A blank experiment, without NLRP1 and MDP (black diamonds) and a positive control with DnaK (black stars) were also performed. (C) Size exclusion chromatography (Superose 6 10/300 column) of NLRP1(227–990) in absence (black solid line), in presence of MDP and ATP (grey dotted line) and in presence of MDP and a non-hydrolysable ATP analogue (grey solid line). Black arrow heads indicate the retention volumes of molecular weight standards.

To characterise the nucleotide bound to NLRP1 an HPLC analysis was carried out using purified NLRP1(227–990). This analysis system clearly distinguishes between ADP and ATP, which have different retention times (Fig 3A, grey solid line). The HPLC profile of the NLRP1 sample clearly shows that the main nucleotide bound to the protein is ATP with a small amount of ADP. Integration of the two peak areas suggests that more than 85% of the protein sample is bound to ATP while the remaining 15% is bound to ADP. Similar results were obtained for the other fragments (data not shown). The malachite green assay was used to analyse the ability of NLRP1 (227–990) to hydrolyse ATP. Interestingly, the protein did not show any signs of hydrolysis activity (Fig 3B, black squares) even after long incubation times (5 hours) (data not shown). Importantly, the addition of MDP, which is suggested to activate NLRP1, did not induce ATP hydrolysis (Fig 3A, black triangles). Taken together, these results indicate that the construct spanning residues 227 up to 990 containing the NACHT-LRR domain of NLRP1 is not able to hydrolyse ATP even in the presence of MDP.

### MDP does not promote oligomerization of NLRP1

MDP has been suggested to be the signal that, together with ATP, activates NLRP1 oligomerization by binding its LRR domain. In order to test this model we performed size exclusion chromatography of protein samples incubated with MDP and ATP or ATPγS. The final concentration of protein used was 0.5 mg/mL, while MDP was 0.2 mg/mL (corresponding to a NLRP1:MDP molar ratio of about 1:100) and ATP (or ATPγS) was 1 mM. The samples were incubated at room temperature for about 3–4 hours and then loaded onto a Superose 6 10/300 column. As shown in Fig 3C, all the samples migrate as a single species with retention volumes that are very similar to the one observed for the NLRP1(227–990) in absence of both MDP and ATP. No high molecular weight species were detected in this experiment suggesting that MDP and ATP alone are not capable of promoting NLRP1(227–990) oligomerization.

### SAXS analysis

**Fig 4:**
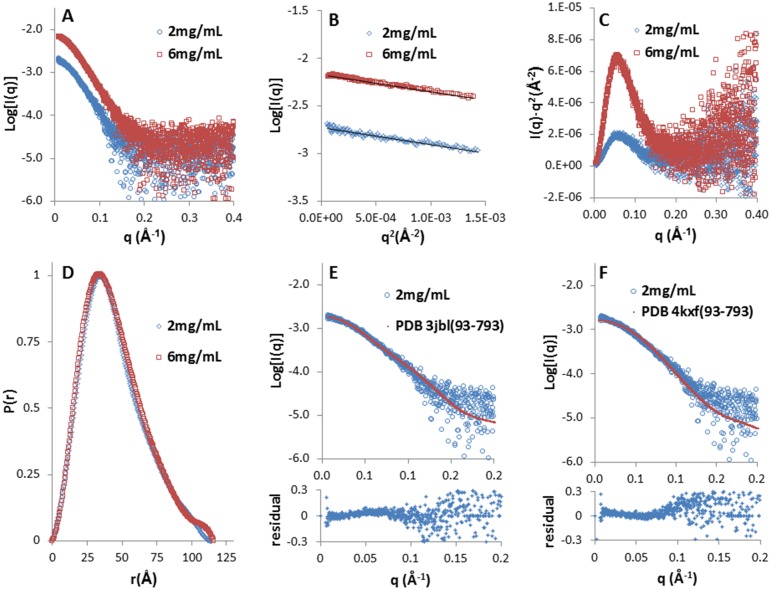
SEC-SAXS analysis of NLRP1(227–990). (A) Scattering data obtained on two protein samples with concentrations of 2 mg/mL (open circles) and 6 mg/mL (open squares) respectively. (B) The linear low-q regions of the scattering curves used for the Guinier analysis. (C) Kratky analysis performed on the data sets at two different concentrations. (D) Normalized inter-atomic pairwise distribution function, P(r), calculated for the two concentrations used in the analysis, showing a maximum particle size of about 115 Å. (E and F) Fitting of the experimental SAXS curve at 2 mg/mL (open circle) with the structure of NLRC4 in two different conformations, open conformation (derived from PDB 3jbl) and closed conformation (derived from PDB 4kxf); the residual difference between the experimental and the calculated values of Log[I(q)] are reported for both fits. In both cases the fitting structures were truncated to residues 93–793 which represent the region that is homologous to the NLRP1 construct used for the SAXS analysis.

Currently no structure is available for fragments of NLRP1 containing multiple domains. To gain structural insight for NLRP1(227–990), we performed small-angle X-ray scattering experiments and calculated its low resolution molecular envelope. SEC-SAXS data at two concentrations (2 mg/mL and 6 mg/mL) are reported in Fig 4A.

Data collection details and parameters from the structural analysis of the scattering curves are reported in Table 1. SAXS data analysis (Fig 4B and 4D) provides a value for the Rg of about 35 Å and a maximum particle dimension derived from the distance distribution function P(r) of around 115 Å (Fig 4D). The anisometric shape of the distance distribution function is indicative of an elongated particle shape with the Kratky plot indicating a system with minor flexibility features (Fig 4C). Three different methods were used to evaluate the SAXS-derived molecular weight. The first approach made use of the approximation that the molecular weight (in Da) is of the order of Vporod(Å^3^)/1.6, this approach produces a value of 87000 Da and 79000 Da for the 2 mg/mL and 6 mg/mL curves respectively. A second evaluation of the molecular weight was performed using the program SAXSMoW (http://www.if.sc.usp.br/~saxs/) obtaining values of 93000 Da and 98000 Da, respectively. A third evaluation was done using BSA as a standard and calculating the molecular weight as [Vporod(NLRP1)·Mw(BSA)]/Vporod(BSA), Vporod of BSA was evaluated from BSA SAXS experiment using 66.2 kDa as the protein molecular weight (Mw(BSA)). The last approach produced values of 99000 Da and 91000 Da for the lower and higher concentration respectively. The three methods all produce values that are close to the molecular weight of the monomeric NLRP1 suggesting that in solution the protein is present as a monomeric species. This result agrees well with the size exclusion chromatography profiles reported in Figs 2A and 3C.

**Table 1:** SAXS-derived parameters for NLRP1(227–990).

**Data-collection Parameters**		
Instrument	SEC-SAXS at B21 Diamond Light source	
SEC column	Superdex 200 (GE Healthcare)	
*q* range (Å^-1^)	0.0022–0.42	
Temperature (°C)	25°C	
Sample Concentration (before SEC)	2mg/mL	6mg/mL
**Structural parameters**		
I(0) (cm^-1^/absorbance) [from P(r)]	0.0018±0.0001	0.0067±0.0002
R_g_ (Å) [from P(r)]	34.6±0.4	35.0±0.1
I(0) (cm^-1^/absorbance) (from Guinier)	0.0018±0.0001	0.0066±0.0002
R_g_ (Å) (from Guinier)	34.1±0.4	34.8±0.2
D_max_ (Å)	114	115
Porod Volume estimate (Å^3^)	139208	127250
χ^2^ ^a^	0.96	1.02
Normalised Spatial Discrepancy	0.57±0.03	0.61±0.03
**SAXS derived Molecular Mass**		
From Porod Volume (V_Porod_/1.6) (Da)	87000	79000
SAXSMoW (Da)^b^	101000	98000
From BSA as standard	99000	91000
Molecular mass from sequence (kDa)	89.5	
**Software employed**		
Primary data reduction	SCATTER	
Data processing	ATSAS	
*Ab initio* analysis	DAMMIF	
Validation and averaging	DAMAVER	
Computation of model intensities	CRYSOL	
Three-dimensional graphic representation	PyMOL	

^a^value derived from best DAMMIF model

^b^ http://www.if.sc.usp.br/~saxs/

**Fig 5:**
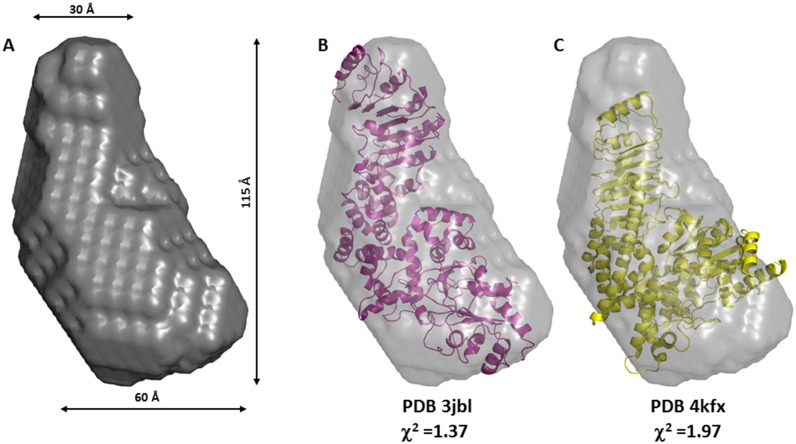
SAXS-derived envelope of NLRP1(227–990) and comparison to the open and closed conformations of NLRC4. (A) SAXS-derived envelope for NLRP1(227–990) calculated from the SAXS data obtained at 2 mg/mL, the maximum dimension (Dmax) and the width of the upper and lower lobes are also reported. (B and C) Structural fitting of the structures of NLRC4 spanning residues 93 to 793 in an open conformation (PDB 3jbl, χ^2^ of 1.37, in magenta) and in a closed conformation (PDB 4kxf, χ^2^ of 1.97, in yellow) into the NLRP1 envelope.

The *ab-inito* low resolution molecular shape calculated by DAMMIF shows an elongated envelope where two lobes are clearly distinguishable (Fig 5A), a larger one with a width of *ca*. 60 Å and a smaller one with a width of *ca* 30 Å. To date, the closest structural homologue for human NLRP1(227–990) is represented by the structure of mouse NLRC4. Two possible conformations have been recently reported for NLRC4: a closed conformation bound to ADP (PDB 4kxf) [42] and an open conformation that self-associate to form an inflammasome complex (PDBs 3jbl, 5aj2) [27–29]. Both structures lack the N-terminal CARD domain, and span residues 93–1024 including both the NACHT and LRR domain. In contrast to NLRP1(227–990), NLRC4 has a much longer LRR domain and a structural alignment of the LRR domain of NLRC4 with that of the human NLRP1 (PDB 4im6) [34] shows that the residues 582–793 from NLRC4 overlap with the residues 792–990 in human NLRP1, with a Cα RMSD of 3.4 Å. Therefore we used the coordinates of fragment 93–793 of NLRC4 to evaluate its solution scattering profile and compared it to the experimental curve for NLRP1. The fitting of the NLRP1 SAXS data, measured at 2 mg/mL, with the structures of the open and closed conformations of NLRC4 are reported in Figs 4E and 4F and 5B and 5C. Interestingly, the open conformation produces a better fit (χ^2^ = 1.37) when compared to the closed conformation (χ^2^ = 1.97) suggesting that the NLRP1 construct used in this study adopts an extended conformation that is reminiscent of the open conformation of NLRC4 in the inflammasome complex. The same analysis carried out on the SAXS data obtained at higher concentration produced almost identical results (data not shown).

## Discussion

NLRP1 is one of the best studied NLRs and although a plethora of data on its biological function are available, many questions regarding its mechanism of activation remain unanswered. The only current activation model for NLRP1, largely based on an *in vitro* study carried out with a version of the enzyme that lacks the part of the C-terminus encoded by exon-14, suggests that the molecule oligomerizes into a ring-shaped complex upon activation by ATP and MDP [33]. However, our data together with a number of other studies cannot be reconciled with the above model and hence the true activation mechanism of human NLRP1 remains unclear.

When we embarked on this project it quickly became apparent that full length NLRP1 is unstable, poorly soluble and has a tendency to aggregate, features that have heavily hampered biophysical studies so far and likely explain some of the current inconsistencies on the NLRP1 activation mechanism found in the literature. To overcome these challenges, we dissected NLRP1 into its constitutive domains in order to obtain protein constructs that could be purified to homogeneity. Interestingly, apart from the pyrin, CARD and LRR domains, for which structures are available, neither the NACHT domain, nor the FIIND domain could be produced in isolation. However, a construct containing both, the NACHT and the LRR domain, was found to be soluble when produced in insect cells, and could be purified to homogeneity. The observation that the NACHT domain can only be produced when the LRR domain is also present suggests that the two domains stabilise each other, probably by sharing an interface. This construct was found to be monomeric in solution, constitutively bound to ATP and devoid of any significant ATP hydrolysis activity. Moreover, when MDP and ATP were added in large excess to the recombinant protein no high molecular species were detected by size exclusion chromatography, suggesting that MDP is not able to promote oligomerization of the construct. Small-angle X-ray scattering data allowed us to obtain a low resolution three-dimensional model that suggests that the molecule in solution behaves as an elongated particle. The SAXS-derived envelope is not consistent with an NLRC4-like closed conformation where the NACHT domain is closed around the ADP nucleotide molecule and the LRR folds back onto it. In contrast, the SAXS data agree well with the structure of NLRC4 in an open conformation as observable in the NLRC4-inflammasome structure.

Since our characterization suggests that NLRP1 shares some similarities with the open conformation of NLRC4 we analysed our data in light of the recently described NLRC4 oligomerization mechanism [27–29]. NLRC4 forms oligomers through the action of a second NLR protein that belongs to the NAIP family. The NAIP protein functions as a sensing element that is activated by a specific bacterial PAMP; the interaction between the activated NAIP and the closed NLRC4 promotes a conformational change that converts NLRC4 into an open state. At this point the oligomerization carries on in a sort of prion-like self-propagating mode where the exposed surface on the open NLRC4 forces other molecules from a closed state into an open state. According to this model, the conformational change is coupled with oligomerization and specific residues were identified as key stabilising elements for the oligomeric state. Surprisingly these residues are not conserved across the NLR family suggesting that the reported mechanism could be specific for NLRC4 and not a generally conserved one. This would imply that different NLRs may adopt different oligomerization mechanisms and indeed this could be the case for NLRP1 for which the ATP-bound open conformation is not sufficient to promote its oligomerization.

A NACHT-domain-containing protein, constitutively bound to ATP and lacking ATP hydrolysis activity is not a novelty. In fact, CED-4 (Cell death protein 4) from *C*. *elegans*, a distant homolog of NLRP1 that plays a key role in programmed cell death, and only contains a CARD and a NACHT domain, is constitutively bound to ATP [43,44]. The triphosphate nucleotide is deeply buried into the structure so that no general base can activate a water molecule and start hydrolysis [45]. CED-4 is kept in an off-state by interacting with CED-9 and the inhibition of this interaction promotes CED-4 oligomerization. On the basis of our results, it is tempting to speculate that NLRP1 might work in a similar way to CED-4 and that the LRR domain may play an equivalent role to the inhibitory molecule CED-9. Upon interaction with a specific activator, which is likely to be different from MDP, the LRR could change its conformation and dissociate from the NACHT domain to allow its oligomerization.

At present it is not known if any of the other domains present, especially the FIIND and C-terminal CARD that are specific to NLRP1, may contribute to ligand sensing and/or oligomerization. Before these questions can be answered it will first be necessary to develop novel methods that allow the production and stabilisation of larger fragments of NLRP1 and render this enigmatic protein accessible to a more detailed structural and biochemical characterization. In conclusion, our study provides new insights that deepen our understanding of NLRP1 and it sheds some light onto its mechanism of activation by revealing a new level of complexity that was unexpected on the basis of previous reports.

## Supporting Information

S1 Fig Multiple sequence alignment of human NLRP1 with human NLRs and NLRP1 sequences from *Mus muculus* and *Rattus morvegicus*. Full length protein sequences for human NLRP1, *Mus muculus* NLRP1a and NLRP1b, *Rattus morvegicus* NLRP1 and the 21 remaining human NLRs were aligned using the Clustal Omega server (http://www.ebi.ac.uk/Tools/msa/clustalo/). The alignment was displayed using Jalview (http://www.jalview.org/) and the residues are shaded according to the percentage of identity. A red box indicates the region of conservation that common to all the sequences. This region spans the NACHT and the LRR domains, a green box indicates an extra region of conservation that is present in the C-terminal part of the NLRP1 sequences, and this region spans the FIIND and the CARD domain.
(DOCX)

S1 Table List of the human NLRP1 constructs that were tested for soluble expression in *E*. *Coli* and insect cells. (DOCX)

## References

[1] Nurnberger T, Brunner F, Kemmerling B, Piater L (2004) Innate immunity in plants and animals: striking similarities and obvious differences. Immunol Rev 198: 249–266. 10.1111/j.0105-2896.2004.0119.x 15199967

[2] Hoffmann JA, Kafatos FC, Janeway CA, Ezekowitz RA (1999) Phylogenetic perspectives in innate immunity. Science 284: 1313–1318. 10.1126/science.284.5418.1313 10334979

[3] Takeuchi O, Akira S (2010) Pattern recognition receptors and inflammation. Cell 140: 805–820. 10.1016/j.cell.2010.01.022 20303872

[4] Medzhitov R, Janeway CA Jr. (1997) Innate immunity: impact on the adaptive immune response. Curr Opin Immunol 9: 4–9. 10.1016/S0952-7915(97)80152-5 9039775

[5] Janeway CA Jr., Medzhitov R (2002) Innate immune recognition. Annu Rev Immunol 20: 197–216. 10.1146/annurev.immunol.20.083001.084359 11861602

[6] Kufer TA, Fritz JH, Philpott DJ (2005) NACHT-LRR proteins (NLRs) in bacterial infection and immunity. Trends Microbiol 13: 381–388. 10.1016/j.tim.2005.06.004 15994078

[7] Proell M, Gerlic M, Mace PD, Reed JC, Riedl SJ (2013) The CARD plays a critical role in ASC foci formation and inflammasome signalling. The Biochemical journal 449: 613–621. 10.1042/BJ20121198 23110696 PMC3966062

[8] Le HT, Harton JA (2013) Pyrin- and CARD-only Proteins as Regulators of NLR Functions. Frontiers in immunology 4: 275 10.3389/fimmu.2013.00275 24062743 PMC3775265

[9] Lechtenberg BC, Mace PD, Riedl SJ (2014) Structural mechanisms in NLR inflammasome signaling. Current opinion in structural biology 29: 17–25. 10.1016/j.sbi.2014.08.011 25201319 PMC4268015

[10] Rosenstiel P, Till A, Schreiber S (2007) NOD-like receptors and human diseases. Microbes and infection / Institut Pasteur 9: 648–657. 10.1016/j.micinf.2007.01.015 17376727

[11] Inohara, Chamaillard, McDonald C, Nuñez G (2005) NOD-LRR proteins: role in host-microbial interactions and inflammatory disease. Annual review of biochemistry 74: 355–383. 10.1146/annurev.biochem.74.082803.133347 15952891

[12] Martinon F, Mayor A, Tschopp J (2009) The inflammasomes: guardians of the body. Annual review of immunology 27: 229–265. 10.1146/annurev.immunol.021908.132715 19302040

[13] Mariathasan S, Monack DM (2007) Inflammasome adaptors and sensors: intracellular regulators of infection and inflammation. Nature reviews Immunology 7: 31–40. 10.1038/nri1997 17186029

[14] Martinon F, Burns K, Tschopp J (2002) The inflammasome: a molecular platform triggering activation of inflammatory caspases and processing of proIL-beta. Molecular cell 10: 417–426. 10.1016/S1097-2765(02)00599-3 12191486

[15] Wen H, Miao EA, Ting JPY (2013) Mechanisms of NOD-like receptor-associated inflammasome activation. Immunity 39: 432–441. 10.1016/j.immuni.2013.08.037 24054327 PMC3835203

[16] Ting JPY, Duncan JA, Lei Y (2010) How the noninflammasome NLRs function in the innate immune system. Science 327: 286–290. 10.1126/science.1184004 20075243 PMC3943909

[17] Strober W, Murray PJ, Kitani A, Watanabe T (2006) Signalling pathways and molecular interactions of NOD1 and NOD2. Nature reviews Immunology 6: 9–20. 10.1038/nri1747 16493424

[18] Hasegawa M, Fujimoto Y, Lucas PC, Nakano H, Fukase K, et al (2008) A critical role of RICK/RIP2 polyubiquitination in Nod-induced NF-kappaB activation. The EMBO journal 27: 373–383. 10.1038/sj.emboj.7601962 18079694 PMC2234345

[19] Man SM, Kanneganti T-D (2015) Regulation of inflammasome activation. Immunological reviews 265: 6–21. 10.1111/imr.12296 25879280 PMC4400844

[20] Latz E, Xiao TS, Stutz A (2013) Activation and regulation of the inflammasomes. Nature reviews Immunology 13: 397–411. 10.1038/nri3452 23702978 PMC3807999

[21] Williams TM, Leeth RA, Rothschild DE, Coutermarsh-Ott SL, McDaniel DK, et al (2015) The NLRP1 inflammasome attenuates colitis and colitis-associated tumorigenesis. Journal of immunology 194: 3369–3380. 10.4049/jimmunol.1402098 25725098 PMC4369420

[22] Nunes T, de Souza HS (2013) Inflammasome in intestinal inflammation and cancer. Mediators Inflamm 2013: 654963 10.1155/2013/654963 23606794 PMC3625567

[23] Bleda S, de Haro J, Varela C, Esparza L, Ferruelo A, et al (2014) NLRP1 inflammasome, and not NLRP3, is the key in the shift to proinflammatory state on endothelial cells in peripheral arterial disease. International journal of cardiology 172: e282–284. 10.1016/j.ijcard.2013.12.201 24439873

[24] Allen IC (2014) Non-Inflammasome Forming NLRs in Inflammation and Tumorigenesis. Front Immunol 5: 169 10.3389/fimmu.2014.00169 24795716 PMC4001041

[25] Reubold TF, Wohlgemuth S, Eschenburg S (2011) Crystal structure of full-length Apaf-1: how the death signal is relayed in the mitochondrial pathway of apoptosis. Structure 19: 1074–1083. 10.1016/j.str.2011.05.013 21827944

[26] Riedl SJ, Li W, Chao Y, Schwarzenbacher R, Shi Y (2005) Structure of the apoptotic protease-activating factor 1 bound to ADP. Nature 434: 926–933. 10.1038/nature03465 15829969

[27] Hu Z, Zhou Q, Zhang C, Fan S, Cheng W, et al (2015) Structural and biochemical basis for induced self-propagation of NLRC4. Science 350: 399–404. 10.1126/science.aac5489 26449475

[28] Zhang L, Chen S, Ruan J, Wu J, Tong AB, et al (2015) Cryo-EM structure of the activated NAIP2-NLRC4 inflammasome reveals nucleated polymerization. Science 350: 404–409. 10.1126/science.aac5789 26449474 PMC4640189

[29] Diebolder CA, Halff EF, Koster AJ, Huizinga EG, Koning RI (2015) Cryoelectron Tomography of the NAIP5/NLRC4 Inflammasome: Implications for NLR Activation. Structure 23: 2349–2357. 10.1016/j.str.2015.10.001 26585513

[30] Chavarria-Smith J, Vance RE (2015) The NLRP1 inflammasomes. Immunol Rev 265: 22–34. 10.1111/imr.12283 25879281

[31] Finger JN, Lich JD, Dare LC, Cook MN, Brown KK, et al (2012) Autolytic proteolysis within the function to find domain (FIIND) is required for NLRP1 inflammasome activity. The Journal of biological chemistry 287: 25030–25037. 10.1074/jbc.M112.378323 22665479 PMC3408201

[32] D'Osualdo A, Weichenberger CX, Wagner RN, Godzik A, Wooley J, et al (2011) CARD8 and NLRP1 undergo autoproteolytic processing through a ZU5-like domain. PloS one 6: e27396 10.1371/journal.pone.0027396 22087307 PMC3210808

[33] Faustin B, Lartigue L, Bruey J-M, Luciano F, Sergienko E, et al (2007) Reconstituted NALP1 inflammasome reveals two-step mechanism of caspase-1 activation. Molecular cell 25: 713–724. 10.1016/j.molcel.2007.01.032 17349957

[34] Reubold TF, Hahne G, Wohlgemuth S, Eschenburg S (2014) Crystal structure of the leucine-rich repeat domain of the NOD-like receptor NLRP1: implications for binding of muramyl dipeptide. FEBS letters 588: 3327–3332. 10.1016/j.febslet.2014.07.017 25064844

[35] Jin T, Curry J, Smith P, Jiang J, Xiao TS (2013) Structure of the NLRP1 caspase recruitment domain suggests potential mechanisms for its association with procaspase-1. Proteins 81: 1266–1270. 10.1002/prot.24287 23508996 PMC3860829

[36] Hiller S, Kohl A, Fiorito F, Herrmann T, Wider G, et al (2003) NMR structure of the apoptosis- and inflammation-related NALP1 pyrin domain. Structure 11: 1199–1205. 10.1016/j.str.2003.08.009 14527388

[37] Zhao Y, Chapman DAG, Jones IM (2003) Improving baculovirus recombination. Nucleic acids research 31: E6–6. 10.1093/nar/gng006 12527795 PMC140531

[38] Petoukhov MV, Franke D, Shkumatov AV, Tria G, Kikhney AG, et al (2012) New developments in the ATSAS program package for small-angle scattering data analysis. J Appl Crystallogr 45: 342–350. 10.1107/S0021889812007662 25484842 PMC4233345

[39] Franke D, Svergun DI (2009) DAMMIF, a program for rapidab-initioshape determination in small-angle scattering. J ApplCryst 42: 342–346. 10.1107/S0021889809000338 27630371 PMC5023043

[40] Volkov VV, Svergun DI (2003) Uniqueness of ab initio shape determination in small-angle scattering. J Appl Cryst 36: 860–864. 10.1107/S0021889809000338 PMC5023043 27630371

[41] Svergun D, Barberato C, Koch MHJ (1995) CRYSOL—a program to evaluate X-ray solution scattering of biological macromolecules from atomic coordinates. J Appl Cryst 28: 768–773.

[42] Hu Z, Yan C, Liu P, Huang Z, Ma R, et al (2013) Crystal structure of NLRC4 reveals its autoinhibition mechanism. Science 341: 172–175. 10.1126/science.1236381 23765277

[43] Yan N, Chai J, Lee ES, Gu L, Liu Q, et al (2005) Structure of the CED-4-CED-9 complex provides insights into programmed cell death in Caenorhabditis elegans. Nature 437: 831–837. 10.1038/nature04002 16208361

[44] Yan N, Xu Y, Shi Y (2006) 2:1 Stoichiometry of the CED-4-CED-9 complex and the tetrameric CED-4: insights into the regulation of CED-3 activation. Cell Cycle 5: 31–34. 10.4161/cc.5.1.2263 16294007

[45] Huang W, Jiang T, Choi W, Qi S, Pang Y, et al (2013) Mechanistic insights into CED-4-mediated activation of CED-3. Genes & development 27: 2039–2048. 10.1101/gad.224428.113 24065769 PMC3792479

