# Supplementary material for: The Biophysical Characterisation and SAXS Analysis of Human NLRP1 Uncover a New Level of Complexity of NLR Proteins
Source: PLoS One. 2016 Oct 11;11(10):e0164662. doi: 10.1371/journal.pone.0164662 (PMC5058472; doi:10.1371/journal.pone.0164662)
Supplement: S1 Fig — Full length protein sequences for human NLRP1, Mus muculus NLRP1a and NLRP1b, Rattus morvegicus NLRP1 and the 21 remaining human NLRs were aligned using the Clustal Omega server (http://www.ebi.ac.uk/Tools/msa/clustalo/). The alignment was displayed using Jalview (http://www.jalview.org/) and the residues are shaded according to the percentage of identity. A red box indicates the region of conservation that common to all the sequences. This region spans the NACHT and the LRR domains, a green box indicates an extra region of conservation that is present in the C-terminal part of the NLRP1 sequences, and this region spans the FIIND and the CARD domain. (DOCX) [file pone.0164662.s001.docx]

**S1 Fig:** Multiple sequence alignment of human NLRP1 with human NLRs and NLRP1 sequences from *Mus muculus* and *Rattus morvegicus*. A red box indicates the region of conservation that common to all the sequences. This region spans the NACHT and the LRR domains, a green box indicates an extra region of conservation that is present in the C-terminal part of the NLRP1 sequences, and this region spans the FIIND and the CARD domain.
