# Supplementary material for: The Biophysical Characterisation and SAXS Analysis of Human NLRP1 Uncover a New Level of Complexity of NLR Proteins
Source: PLoS One. 2016 Oct 11;11(10):e0164662. doi: 10.1371/journal.pone.0164662 (PMC5058472; doi:10.1371/journal.pone.0164662)
Supplement: S1 Table — (DOCX) [file pone.0164662.s002.docx]

**S1 Table. List of the human NLRP1 constructs that were tested for soluble expression in E. Coli and insect cells.**

| **Construct boundaries** | **Domains** | **Expression system** | **solubility** | **Yield of recombinant protein after purification** |
| --- | --- | --- | --- | --- |
| 266-561 | NACHT(NBD) | E.Coli | insoluble | - |
| 266-642 | NACHT(NBD-WH) | E.Coli | insoluble | - |
| 266-699 | NACHT(NBD-WH-SH) | E.Coli | insoluble | - |
| 291-561 | NACHT(NBD) | E.Coli | insoluble | - |
| 291-642 | NACHT(NBD-WH) | E.Coli | insoluble | - |
| 291-699 | NACHT(NBD-WH-SH) | E.Coli | insoluble | - |
| 258-706 | NACHT(NBD-WH-SH) | E.Coli | insoluble | - |
| 258-715 | NACHT(NBD-WH-SH) | E.Coli | insoluble | - |
| 258-725 | NACHT(NBD-WH-SH) | E.Coli | insoluble | - |
| 258-742 | NACHT(NBD-WH-SH) | E.Coli | insoluble | - |
| 258-761 | NACHT(NBD-WH-SH) | E.Coli | insoluble | - |
| 258-771 | NACHT(NBD-WH-SH) | E.Coli | insoluble | - |
| 266-706 | NACHT(NBD-WH-SH) | E.Coli | insoluble | - |
| 266-715 | NACHT(NBD-WH-SH) | E.Coli | insoluble | - |
| 266-725 | NACHT(NBD-WH-SH) | E.Coli | insoluble | - |
| 266-742 | NACHT(NBD-WH-SH) | E.Coli | insoluble | - |
| 266-761 | NACHT(NBD-WH-SH) | E.Coli | insoluble | - |
| 266-771 | NACHT(NBD-WH-SH) | E.Coli | insoluble | - |
| 266-638 | NACHT(NBD-WH) | E.Coli | insoluble | - |
| 266-732 | NACHT(NBD-WH-SH) | E.Coli | insoluble | - |
| 541-989 | NACHT(WH-SH)-LRR | E.Coli | insoluble | - |
| 656-989 | NACHT(SH)-LRR | E.Coli | insoluble | - |
| 1004-1217 | FIIND | E.Coli | insoluble | - |
| 1004-1355 | FIIND | E.Coli | insoluble | - |
| 791-1217 | LRR-FIIND | E.Coli | insoluble | - |
| 791-1355 | LRR-FIIND | E.Coli | insoluble | - |
| 1-1473 | PyrinNACHT-LRR-FIIND-CARD | Insect cells | Soluble/proteolytically degraded | - |
| 1-990 | Pyrin-NACHT-LRR | Insect cells | Soluble/proteolytically degraded | ~ 0.03mg/L of culture |
| 93-990 | NACHT-LRR | Insect cells | Soluble/proteolytically degraded | ~ 0.04mg/L of culture |
| 162-990 | NACHT-LRR | Insect cells | soluble | ~ 0.1 mg/L of culture |
| 193-990 | NACHT-LRR | Insect cells | soluble | ~ 0.5 mg/L of culture |
| 227-990 | NACHT-LRR | Insect cells | soluble | ~ 2 mg/L of culture |
| 247-990 | NACHT-LRR | Insect cells | soluble | ~ 0.7 mg/L of culture |
| 258-990 | NACHT-LRR | Insect cells | soluble | ~ 0.5 mg/L of culture |
| 227-1355 | NACHT-LRR-FIIND | Insect cells | Soluble/proteolytically degraded | - |
|  | | | | |
